# An Update on the Cosmetic Use of Botulinum Toxin: The Pattern of Practice among Korean Dermatologists

**DOI:** 10.3390/toxins14050329

**Published:** 2022-05-04

**Authors:** Nark-Kyoung Rho, Kwang-Ho Han, Hei-Sung Kim

**Affiliations:** 1 Leaders Aesthetic Laser and Cosmetic Surgery Center, Seoul 06014, Korea; rhonark@hanmail.net; 2 Department of Dermatology, Sungkyunkwan University School of Medicine, Seoul 06355, Korea; 3 Korean Society for Anti-Aging Dermatology (KAAD), 385 Seongnam-daero, Seoul 13555, Korea; drhankh@nate.com; 4 Nature Dermatology, Seoul 06055, Korea; 5 Department of Dermatology, Incheon St. Mary’s Hospital, The Catholic University of Korea, Seoul 06591, Korea

**Keywords:** update, cosmetic use, botulinum toxin, practice pattern, Korean dermatologists

## Abstract

The efficacy and safety of botulinum toxin injection have made it a popular aesthetic procedure worldwide. A cross-sectional survey was performed in order to determine the pattern of type A botulinum toxin injections in cosmetic practice, for which an 18-item questionnaire was distributed to dermatologists. A total of 469 Korean board-certified dermatologists participated in the survey, with the following results: the main candidates for type A botulinum toxin injection were individuals in their 40–50 years (46.1%), followed by those in their 20–30 years (33.4%), and people over 60 years of age (20.5%). Overall, the upper face (the glabella, forehead, and crow’s line, in decreasing order) was the most favored area of injection (51%). In contrast, body contouring (i.e., shoulder, calf) and treatment for benign masseter hypertrophy were significantly more popular in the 20–30 years age group than their older counterparts. For wrinkle effacement, the most preferred dilution was 100 units/2.5 mL with isotonic sodium chloride injection (51.2%), and the most often used interval was six months (43.6%). About half (46.3%) of the dermatologists reported the experience of clinical cases which were suspicious of botulinum toxin resistance. Despite this, regarding the choice of the product, type A botulinum toxin products with greater cost-effectiveness were favored over products with a lower risk of antibody formation. Other than its cosmetic usage, botulinum toxin is applied for a variety of skin conditions. Further studies are suggested in order to identify the practice pattern of type A botulinum toxin for therapeutic uses in dermatology, such as hyperhidrosis and rosacea.

## 1. Introduction

Since the first approval of Botox^®^ (Allergan, Irvine, CA, USA) in 1995, type A botulinum toxin (BoNT-A) injections quickly became a key cosmetic procedure in Korean dermatology settings, alongside laser therapy [1]. Looking at our situation in 2021, Korea has a fiercely competitive BoNT-A market with a wide variety of commercial products, including Botox^®^/Vistabel^®^, Dysport^®^/Azzalure^®^ (Galderma, Zählerweg, Switzerland), Botulax^®^ (Hugel, Seoul, Korea), BTX-A^®^ (Lanzhou, China), Coretox^®^ (Medy-Tox, Cheongju, Korea), Innotox^®^ (Medy-Tox, Korea), Meditoxin^®^/Neuronox^®^ (Medy-Tox, Korea), Nabota^®^/Jeuveau^®^ (Daewoong Pharmaceutical, Seoul, Korea), and Xeomin^®^/Bocouture^®^ (Merz Pharma, Frankfurt, Germany) [2]. Considering that only a couple of companies compete worldwide in the botulinum toxin drug market due to difficulties in the toxin’s manufacture and handling, it is surprising that half of the products in the market are from Korea. In addition, the Korean botulinum toxin market is continuously expanding in terms of quantity with the introduction of new products such as Liztox^®^ (Humedix, Seongnam, Korea) and ReNTox^®^ (PharmaResearch Bio, Gangneung, Korea). The price of domestic botulinum toxin products is currently very low compared to the global standard due to competition, which has brought about the effect of popularizing toxin injection. Because Korea is a major manufacturing supplier of botulinum toxin products, it will likely impact the global market.

Apart from this quantitative expansion, continuous progress has been made in refining the treatment methods and expanding the indications (Tables S1 and S2), including cosmetic indications (Table 1, Table 2 and Table 3). A consensus recommendation on the aesthetic usage of BoNT-A in Asians has been published [3] in order to ensure treatment safety and efficacy with BoNT-A injection. While BoNT-A injection is actively practiced in Korea, we know little about its use in real-world cosmetic practice. Because botulinum toxin injection is not a subsidized procedure, Korean National Health Insurance data are unavailable for large-scale epidemiological studies. In order to optimize the current practice with BoNT-A in aesthetic medicine, clinicians need to be aware of the contemporary trends of BoNT-A injections, including patient characteristics, common cosmetic indications, reconstitution methods, and treatment intervals, as well as side effects.

## 2. Materials and Methods

An 18-item questionnaire on the practice pattern of botulinum toxin use was developed by the Korean Society for Anti-aging Dermatology (KAAD) (Table 4). The questionnaire was distributed to 1366 Korean dermatologists who practice in various settings (primary, secondary, and tertiary hospitals). A web-based survey platform (http://www.ozsurvey.co.kr, accessed from 25 February 2019 to 23 March 2019) [4] was adopted. The questionnaire link was sent through mobile phone text messages, and the respondents answered by accessing the site. A total of 469 Korean board-certified dermatologists participated in the survey (response rate: 34.4%), of which 326 answered all of the questions (survey completion) and 143 showed partial response. This survey study was ethically approved by the Institutional Review Board (IRB) of Incheon St. Mary’s Hospital, The Catholic University of Korea (OC21QCSI0131).

## 3. Results

### 3.1. What Is Your Practice Setting?

Of the 469 dermatologists who responded to the questionnaire, 82.3% worked in private clinics and 17.7% worked in secondary or tertiary referral hospitals.

### 3.2. How Long Have You been Practicing Botulinum Toxin Injections?

In total, 52.1% of the survey participants had more than 5 years of experience with botulinum toxin, with 27.1% having experience of more than 10 years. In total, 32.9% of the respondents had 1–5 years of experience, and 15% had practiced botulinum toxin injection for less than a year. 

### 3.3. What Is the Approximate Percentage of Patients Who Visit Your Office for Cosmetic Procedures and for Disease Treatment?

The survey responders attended both to patients with skin disease (53.7%) and to those requesting or in need of cosmetic procedures (46.3%). 

### 3.4. What Is the Patient Proportion, by Age (Years), of Those Who Visit for Cosmetic Procedures?

**Figure 1:**
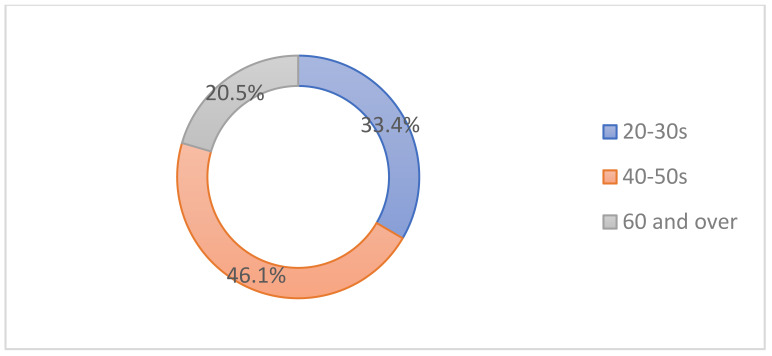
Patient proportion by age for those who visit for cosmetic procedures.

The main candidates were individuals in their 40–50s (46.1%), followed by those in their 20–30s (33.4%), and people over 60 years of age (20.5%) (Figure 1). 

### 3.5. Among Those Who Receive Cosmetic Botulinum Toxin Injections, What Is the Treatment Rate by Site?

**Figure 2:**
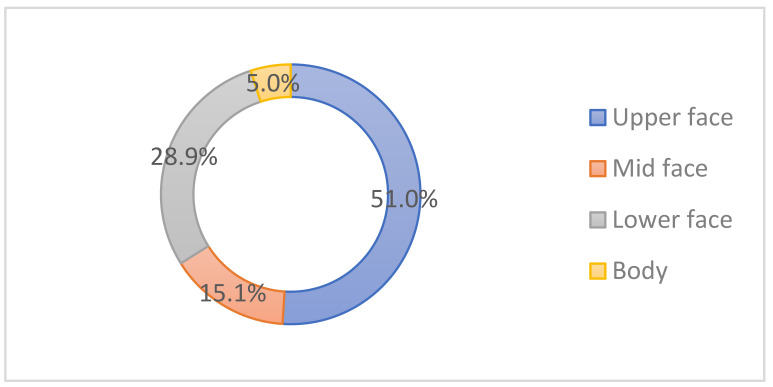
Botulinum toxin treatment rate by site.

Overall, the upper face was the most favored area of injection (51%), followed by the lower face/neck (28.9%), mid-face (15.1%), and body (5%) (Figure 2).

### 3.6. What Are the Three Most Frequent Indications among Patients Who Receive Cosmetic Botulinum Toxin Injections? 

**Figure 3:**
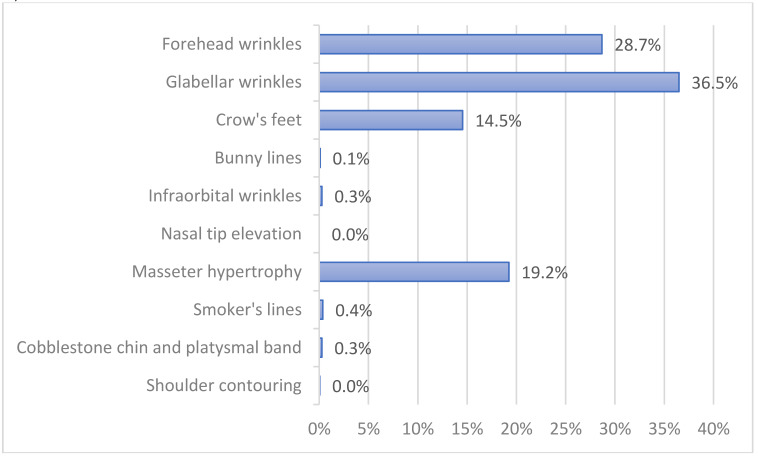
Three most common cosmetic indications for botulinum toxin injection.

Glabellar wrinkles (36.5%), forehead wrinkles (28.7%), masseter hypertrophy (19.2%) and crow’s feet (14.5%) were the most popular sites of botulinum toxin injection (Figure 3).

### 3.7. Among Those in Their 20-30 Years Who Receive Cosmetic Botulinum Toxin Injections, What Is the Rate of the Procedure By Site? 

**Figure 4:**
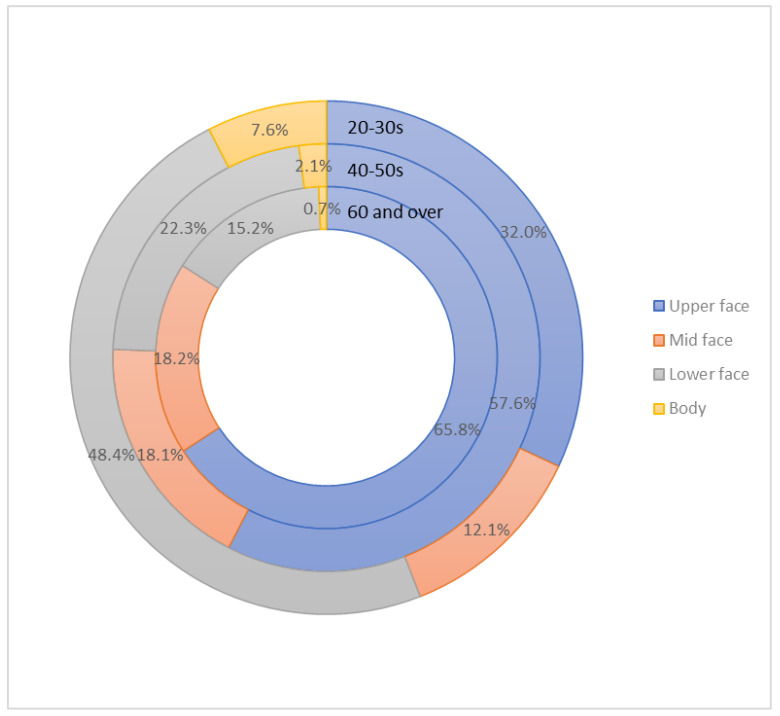
The rate of the procedure by site in the different age groups.

The lower face/neck was the most frequently requested treatment site (48.4%), followed by the upper face (32%), mid-face (12.1%), and body (7.6%) in the 20-30 years age group (Figure 4). 

### 3.8. Among Those in Their 40–50 Years Who Receive Cosmetic Botulinum Toxin Injections, What Is the Rate of the Procedure by Site?

The upper face was the most preferred area of treatment (57.6%), followed by the lower face/neck (22.3%), mid-face (18.1%), and body (2.1%) in the 40s–50s age group (Figure 4). 

### 3.9. Among the Patients Aged 60 Years and Older Who Receive Cosmetic Botulinum Toxin Injections, What Are the Rates of the Procedure by Site?

The upper face was the most popular treatment site (65.8%), followed by the mid-face (18.2%), lower face/neck (15.2%), and body (0.7%) in the group aged 60 years and older (Figure 4). 

### 3.10. Please Rank the Following Indications Based on Their Popularity among Patients Who Receive Cosmetic Botulinum Toxin Injections in the Upper Face

**Figure 5:**
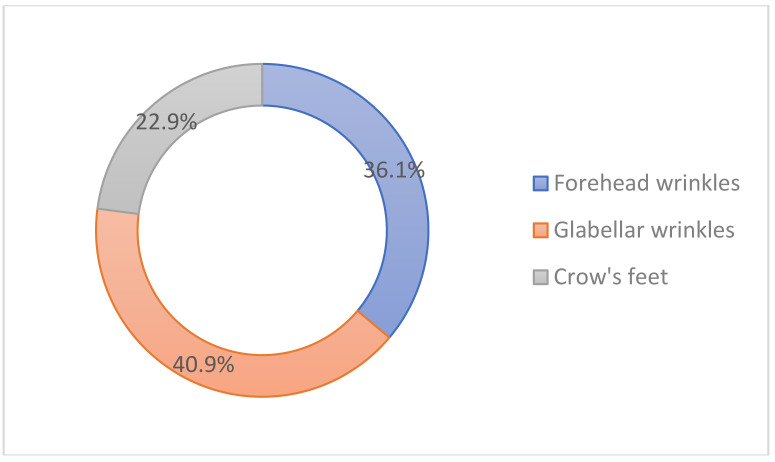
Popular indications for cosmetic botulinum toxin injections in the upper face.

The glabellar wrinkles were the most popular indication (40.9%), followed by the forehead wrinkles (36.1%), and crow’s feet (22.9%) in the upper face (Figure 5).

### 3.11. Please Rank the Following Indications Based on their Popularity among Patients Who Receive Cosmetic Botulinum Toxin Injections in the Midface

**Figure 6:**
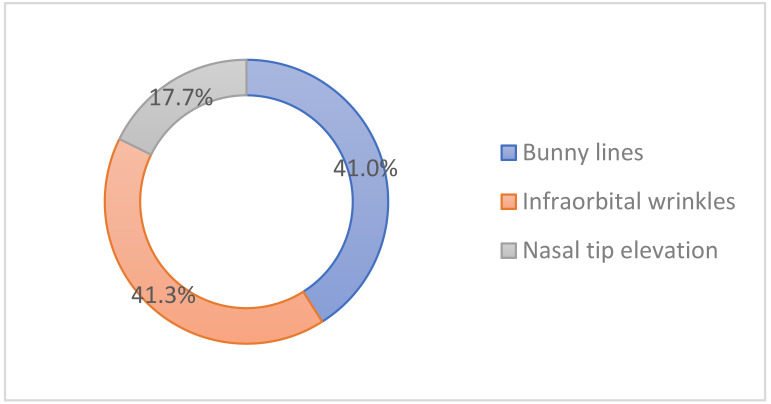
Popular indications for cosmetic botulinum toxin injections in the mid-face.

Infraorbital wrinkles were the most popular indication (41.3%), followed by the bunny lines (41%) and the nasal tip elevation (17.7%) in the mid-face (Figure 6).

### 3.12. Please Rank the Following Indications Based on Their Popularity among Patients Who Receive Cosmetic Botulinum Toxin Injections in the Lower Face/Neck

**Figure 7:**
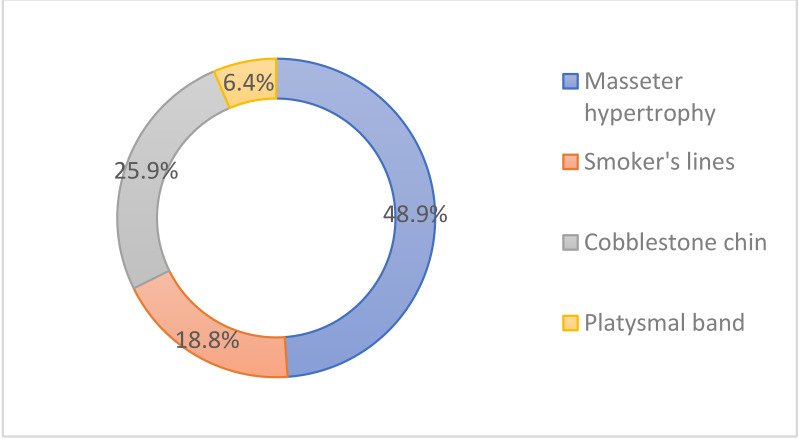
Popular indications for cosmetic botulinum toxin injections in the lower face/neck.

Masseter hypertrophy was the most popular indication (48.9%), followed by cobblestone chin (25.9%), smoker’s lines (18.8%), and platysmal band (6.4%) in the lower face/neck (Figure 7).

### 3.13. Please Rank the Following Indications Based on Their Popularity among Patients Who Receive Cosmetic Botulinum Toxin Injections in the Body (Trunk or Extremities)

**Figure 8:**
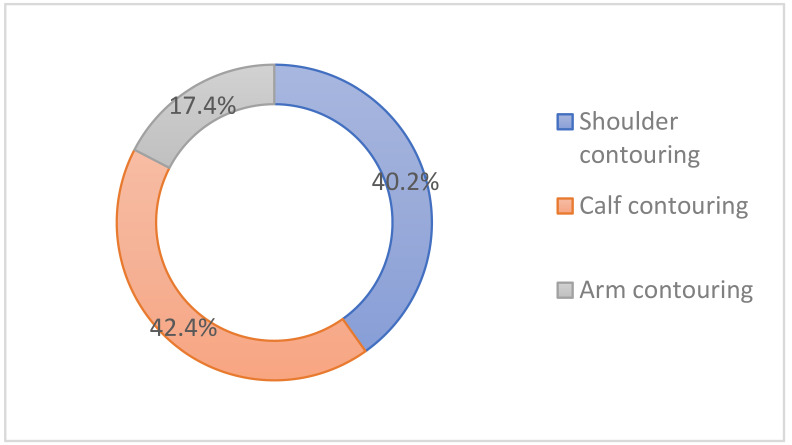
Popular indications for cosmetic botulinum toxin injections in the body.

Calf contouring was the most popular indication (42.4%), followed by shoulder contouring (40.2%) and arm contouring (17.4%) in the body (Figure 8).

### 3.14. When Reconstituting a Botulinum Toxin Product for Wrinkle Effacement, What Amount of Isotonic Saline Do You Add for 100 Units of Type A Botulinum Toxin?

In total, 51.2% of the survey responders opted for 2.5 mL isotonic saline for reconstitution, followed by 4 mL (18.1%), 2 mL (15.6%), 5 mL (12.3%), and 1 mL (2.8%).

### 3.15. Have You Experienced Cases Which were Suspicious of Clinical Resistance to Botulinum Toxin Treatment?

In total, 46.3% of the survey responders replied that they had experienced cases which were suspicious of clinical resistance to botulinum toxin treatment.

### 3.16. What Is Your Routine Treatment Interval for Botulinum Toxin Injections?

In total, 43.6% of the survey responders set the treatment interval at 6 months or more (43.6%), followed by 4 months (23.3%), 5 months (15%), 3 months (16.3%), and 2 months or less (1.8%).

### 3.17. What Factor Do You Consider to Be the Most Important in Choosing a Botulinum Toxin Product?

In total, 72.4% of the survey responders chose reliability (stability, constant potency, therapeutic efficacy) as the most important factor in choosing a product, followed by the price (23.6%), a lower risk of antibody formation (2.5%), and the liquid formulation of the product (1.5%). 

### 3.18. What Commercial Product Do You Use the Most? (Bar Graph/Column Chart)

**Figure 9:**
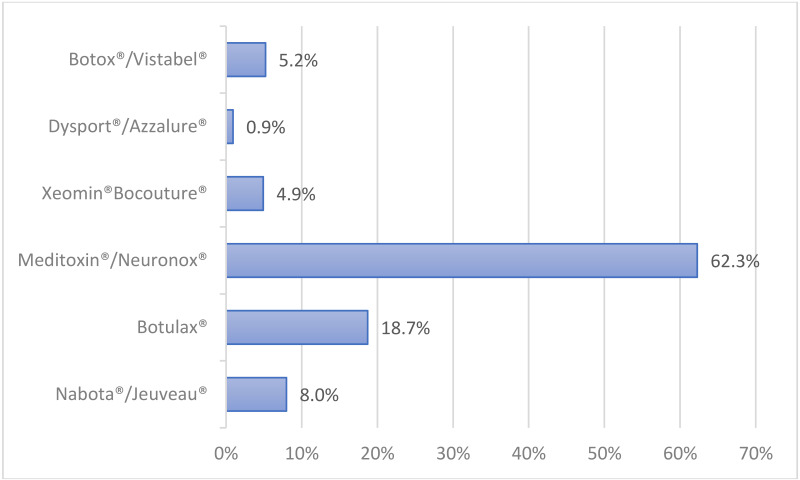
The most favored botulinum toxin products.

Among the survey responders, Meditoxin^®^/Neuronox^®^ (62.3%) was the most favored product, followed by Botulax^®^ (18.7%), Nabota^®^/Jeuveau^®^ (8%), Botox^®^/Vistabel^®^ (5.2%), Xeomin^®^/ Bocouture^®^ (4.9%), and Dysport^®^/Azzalure^®^ (0.9%) (Figure 9).

## 4. Discussion

Based on real-world experiences, the present work is a follow-up to the consensus paper regarding the aesthetic usage of BoNT-A in Asians, which the authors reported several years ago [3]. Compared to other subspecialties, dermatologists have been voted as the most qualified specialist to perform cosmetic BoNT-A injections by primary care physicians [5], which may add validity to this study.

Our survey results reveal that patients over 40 years of age and those in their 20–30s constitute a high proportion of subjects receiving cosmetic BoNT-A injections (33.4%), which displays that there is universal interest among Korean adults in the procedure.

The most sought-after cosmetic indications for BoNT-A injections were the upper facial wrinkles (glabella wrinkles, forehead lines, and crow’s feet, in decreasing order) which are in line with findings from Mass et al. [6] and Hugul et al. [7]. Masseteric hypertrophy was also a preferred indication, which correlates with the high demand for a slim mandible in Koreans [3]. Such popularity may not be seen in Caucasians, as Asians and Caucasians have different beauty ideals in the lower face, i.e., Asians generally prefer a slim lateral jawline, whereas a well-defined mandible is generally favored by Caucasians [8].

Body contouring with botulinum toxin has become increasingly popular in Korea as an off-label indication. Enlarged, muscular calves, shoulders, and upper arms are a unique esthetic concern among many Asian women, especially for the younger generations, and many prefer to slim them down [9,10]. BoNT-A injections have been reported to be an effective and safe option to non-surgically reduce the size and reshape the contour of the calves (gastrocnemius muscles) and the shoulder (trapezius, deltoid, and biceps muscles) [11,12].

As for the reconstitution of a product, one vial of BoNT-A (100 units) was most frequently diluted with 2.5 mL isotonic saline for wrinkle effacement (51.2% of respondents), which is similar to the result from a survey that was performed in 2009 by a Korean BoNT-A manufacturing company [3]. In our previous consensus paper [3], the authors recommended reconstituting a 100-unit vial with 2 mL isotonic saline for practical reasons, as this results in a single scale of an insulin syringe (0.02 mL) being equivalent to 1 unit of BoNT-A, which is more straightforward for beginners when drawing out the toxin. In the present study, BoNT-A dilution with 4 mL saline was also favored, and was used more frequently than 2 mL [3]. Considering that the total number of units of the toxin delivered to the target site is generally more important than the concentration, the reconstitution dosage can be decided based upon the injector’s judgment, planning, and skill [8].

While BoNT-A is considered safe and effective in cosmetic practice, its use has been complicated by neutralizing or non-neutralizing antibodies that can decrease its therapeutic efficacy [13]. In the present survey, nearly half of the respondents suspected to have experienced clinically resistant cases to BoNT-A treatment, which may be surprising surprising considering that relatively low dosages of BoNT-A are injected for cosmetic indications. However, the finding should be interpreted with care as dermatologists who have experienced resistance (not necessarily treatment failure) even in one of the hundreds-thousands of patients he or she treated would have answered “yes”. Our findings are supported by the publication from Park et al. where 57% of Asian-Pacific BoNT-A experts reported to believe that treatment resistance can occur from cosmetic treatment and suspect that some of their patients currently exhibit treatment resistance [14]. 

As for our survey, clinical resistance to BoNT-A was suspected when treatment did not give the same visible results or if the duration of effect was far less compared to previous injections even with an increase in dosage. Although not a routine procedure, Korean dermatologists often perform the frontalis antibody test (FTAT) and in rare cases strongly suspicious of neutralizing antibodies being present (i.e., clinical treatment failure), blood samples are sent out to BoNT-A companies for ELISA. Unfortunately, our questionnaire did not ask if clinical resistance tests such as frontalis antibody test (FTAT) or antibody detection assays [13] were further performed. Interestingly, 19% of Asian-Pacific BoNT-A experts reported to have encountered treatment failure in patients with confirmed neutralizing antibody titer [14] which we hope to examine in our next study. 

It is widely understood that the presence of antibodies is attributed to shorter dosing intervals, higher total dose per injection, and increased amounts of antigenic proteins [13]. In the present survey, the respondents favored a relatively long dosing interval (6 months or more in 43.6% of the respondents), implying that Korean dermatologists tend to avoid too frequent BoNT-A injections, which may cause antibody-induced treatment failure. Interestingly enough many regarded cost-effectiveness to be more important than the lower risk of antibody formation (i.e., formulations without non-toxic accessory proteins) in choosing a BoNT-A product for their daily practice. This finding implies that BoNT-A resistance does not have substantial clinical impact in cosmetic settings. Nevertheless, when antibody-induced resistance is suspected, history of BoNT-A exposure should be thoroughly explored, followed by laboratory investigations according to the algorithm suggested by Srinoulprasert et al. [15]. Inhibition ELISA (detects antibody against all active sites of BoNT-A) and absorption ELISA (detects antibodies against accessory or complexing proteins) as well as functional tests are mentioned in the algorithm [16].

The three most favored BoNT-A products in this survey were all Korean products, suggesting that Korean dermatologists now consider domestic BoTA products to have comparable efficacy to Botox^®^ while having better cost-effectiveness profile. By using novel test methods, Hong et al. [17] recently compared the potency of four widely used BoTA products in Korea to find differences in the total amount of botulinum toxin protein and the cleavage activity of SNAP-25. In terms of potency, Meditoxin^®^/Neuronox^®^ (Medy-Tox, Korea), and Nabota^®^/Jeuveau^®^ (Daewoong Pharmaceutical, Korea) exhibited similar potency with Botox^®^/Vistabel^®^, while Botulax^®^ (Hugel, Korea) showed significantly greater potency per unit than Botox^®^/Vistabel^®^ in vivo and in vitro studies. More scientific studies using reliable test methods would better evaluate and compare various commercial products and thus enable clinicians to provide an optimal outcome. 

The present survey has several limitations. First, we could not identify the use of botulinum toxin in disease conditions of the skin, such as itching, flushing, rosacea, and scars. Secondly, we were unable to stratify treatment indications by gender in the present survey. Also, our results do not provide information on the possible differences in the practice patterns between the Asian and European/US-countries.

## 5. Conclusions

Our results display the universal interest among Korean adults in the procedure, with many receiving cosmetic BoNT-A injections in their 20–30 years. While traditional indications (i.e., wrinkle effacement in the upper face) were popular in all of the age groups, younger individuals received more off-label indications (i.e., masseter hypertrophy and body contouring), which may be unique to Asians due to their different perspectives of beauty. Korean BoNT-A products were favored because the dermatologists opted for cost-effective products, and despite the awareness to BoNT-A resistance, selecting a BoNT-A formulation with less antigenic potential was not prioritized. We hope this study enables clinicians to understand patients’ needs better, and to optimize cosmetic practice with BoNT-A. In order to further elucidate the gender, ethnic, and cultural differences in the cosmetic BoNT-A practice in dermatology, we suggest a comparative study based on a more tailored survey design.

## Figures and Tables

**Table 1:** On-label cosmetic indications for different BoNT-A products available in Korea.

Commercial Name	Manufacturer	Horizontal Forehead Dynamic Lines	Glabellar Dynamic Wrinkles	Lateral Canthal Dynamic Wrinkles
Botox^®^/Vistabel^®^	Allergan (U.S.A.)	□	■	■
Botox^®^/Vistabel^®^50U	Allergan (U.S.A.)	□	■	■
Botulax^®^	Hugel (Korea)	□	■	■
Botulax^®^150U	Hugel (Korea)	□	■	■
Botulax^®^200U	Hugel (Korea)	□	■	■
Botulax^®^300U	Hugel (Korea)	□	□	□
Botulax^®^50U	Hugel (Korea)	□	■	■
BTX-A^®^	Lanzhou (China)	□	□	□
Coretox^®^	Medy-Tox (Korea)	□	■	□
Dysport^®^/Azzalure^®^	Galderma (Switzerland)	□	■	□
Innotox^®^	Medy-Tox (Korea)	□	■	□
Liztox^®^100U	Huons (Korea)	□	■	■
Liztox^®^200U	Huons (Korea)	□	■	■
Liztox^®^50U	Huons (Korea)	□	■	■
Meditoxin^®^/Neuronox^®^	Medy-Tox (Korea)	□	■	■
Meditoxin^®^/Neuronox^®^150U	Medy-Tox (Korea)	□	■	■
Meditoxin^®^/Neuronox^®^200U	Medy-Tox (Korea)	□	■	■
Meditoxin^®^/Neuronox^®^50U	Medy-Tox (Korea)	□	■	■
Nabota^®^/Jeuveau^®^	Daewoong (Korea)	□	■	■
Nabota^®^/Jeuveau^®^150U	Daewoong (Korea)	□	■	■
Nabota^®^/Jeuveau^®^200U	Daewoong (Korea)	□	■	■
Nabota^®^/Jeuveau^®^25U	Daewoong (Korea)	□	■	□
Nabota^®^/Jeuveau^®^50U	Daewoong (Korea)	□	■	■
Xeomin^®^/Bocouture^®^	Merz (Germany)	■	■	■
Xeomin^®^/Bocouture^®^50U	Merz (Germany)	■	■	■

■: On-label indication.

**Table 2:** On-label cosmetic indications for export-only Korean BoNT-A products.

Commercial Name	Manufacturer	Horizontal Forehead Dynamic Lines	Glabellar Dynamic Wrinkles	Lateral Canthal Dynamic Wrinkles
Bienox^®^	BNC Korea (Korea)	□	■	□
Hitox^®^	BMI Korea (Korea)	□	■	□
Inibo^®^	Inibio (Korea)	□	■	□
Jetema The Toxin^®^100U	Jetema (Korea)	□	■	□
Jetema The Toxin^®^200U	Jetema (Korea)	□	■	□
Protoxin^®^100U	Protox (Korea)	□	■	□
ReNTOX^®^100U	PharmaResearch (Korea)	□	■	□
Tyemvers^®^	Chong Kun Dang (Korea)	□	■	□

■: On-label indication.

**Table 3:** Off-label cosmetic indications for BoNT-A products.

**Off-Label Cosmetic Indications for BoNT-A Products**
Bunny lines
Infraorbital wrinkles
Nasal tip elevation
Masseter hypertrophy
Smoker’s lines
Cobblestone chin and platysmal band
Shoulder contouring
Upper arm contouring
Calf contouring

**Table 4:** An 18-item questionnaire on the practice pattern of cosmetic botulinum toxin injections.

**Q1.**	**What is your practice setting?**
□	Private clinic
□	Secondary or tertiary referral hospital
**Q2**	**How long have you been practicing botulinum toxin injections?**
□	Less than 1 year
□	1–5 years
□	5–10 years
□	More than 10 years
**Q3**	**What is the approximate percentage of patients who visit your office for cosmetic procedures and for disease treatment? (the total should be 100)**
□	For cosmetic procedures: _______(%)
□	For disease treatment: _______(%)
**Q4**	**What is the patient proportion, by age (years), of those who visit for cosmetic procedures? (total should be 100)**
□	20–30 years: _______(%)
□	40–50 years: _______(%)
□	60 and over: _______(%)
**Q5**	**Among those who receive cosmetic botulinum toxin injections, what is the treatment rate by site? (total should be 100)**
□	Upper face: _______(%)
□	Midface: _______(%)
□	Lower face/neck: _______(%)
□	Body (trunk or extremities): _______(%)
**Q6**	**What are the three most frequent indications among patients who receive cosmetic botulinum toxin injections?**
□	Forehead wrinkles
□	Glabellar wrinkles
□	Crow’s feet
□	Bunny lines
□	Infraorbital wrinkles
□	Nasal tip elevation
□	Masseter hypertrophy
□	Smoker’s lines
□	Cobblestone chin and platysmal band
□	Shoulder contouring (Trapezius muscle injection)
□	1st: _________________________
□	2nd: _________________________
□	3rd: __________________________
**Q7**	**Among those in their 20–30 years who receive cosmetic botulinum toxin injections, what is the rate of the procedure by site? (total should be 100)**
□	Upper face: _______(%)
□	Mid face: _______(%)
□	Lower face/neck: _______(%)
□	Body: _______(%)
**Q8**	**Among those in their 40–50 years who receive cosmetic botulinum toxin injections, what is the rate of the procedure by site?** **(total should be 100)**
□	Upper face: _______(%)
□	Mid face: _______(%)
□	Lower face/neck: _______(%)
□	Body: _______(%)
**Q9**	**Among the patients aged 60 years and older who receive cosmetic botulinum toxin injections, what is the rate of the procedure by site? (total should be 100)**
□	Upper face: _______(%)
□	Mid face: _______(%)
□	Lower face/neck: _______(%)
□	Body: _______(%)
**Q10**	**Please rank the following indications based on their popularity among patients who receive cosmetic botulinum toxin injections in the upper face.**
□	Forehead wrinkles
□	Glabellar wrinkles
□	Periorbital wrinkles (“crow’s feet lines”)
□	1st: _________________________
□	2nd: _________________________
□	3rd: __________________________
**Q11**	**Please rank the following indications based on their popularity among patients who receive cosmetic botulinum toxin injections in the midface.**
	Nasal wrinkles (“bunny lines”)
□	Infraorbital wrinkles
□	Drooping nasal tip
□	1st: _________________________
□	2nd: _________________________
□	3rd: __________________________
**Q12**	**Please rank the following indications based on their popularity among patients who receive cosmetic botulinum toxin injections in the lower face/neck.**
□	Masseter hypertrophy
□	Vertical perioral wrinkles (“smoker’s lines”)
□	Chin creases (“cobblestone chin”)
□	Platysmal bands
□	1st: _________________________
□	2nd: _________________________
□	3rd: __________________________
**Q13**	**Please rank the following indications based on their popularity among patients who receive cosmetic botulinum toxin injections in the body (trunk or extremities).**
□	Shoulder contouring
□	Calf contouring
□	Upper arm contouring
□	1st: _________________________
□	2nd: _________________________
□	3rd: __________________________
**Q14**	**When reconstituting a botulinum toxin product for wrinkle effacement, what amount of isotonic saline do you add for 100 units of type A botulinum toxin?**
□	1 mL
□	2 mL
□	2.5 mL
□	4 mL
□	5 mL
**Q15**	**Have you experienced cases suspicious of clinical resistance to botulinum toxin treatment?**
□	No
□	Yes
**Q16**	**What is your routine treatment interval for botulinum toxin injections?**
□	2 months (or less)
□	3 months
□	4 months
□	5 months
□	6 months (or more)
**Q17**	**What factor do you consider the most in choosing a botulinum toxin product?**
□	Low price
□	Liquid formulation (pre-reconstituted botulinum toxin)
□	Low risk of antibody formation (low risk of clinical resistance)
□	Reliability (stability-constant potency, therapeutic efficacy)
**Q18**	**What commercial product do you use the most?**
□	Botox^®^ Vistabel^®^ (Allergan, USA)
□	Dysport^®^/Azzalure^®^ (Galderma, Switzerland)
□	Xeomin^®^/ Bocouture^®^ (Merz, Germany)
□	Meditoxin^®^/Neuronox^®^ (Medy-Tox, Korea)
□	Botulax^®^ (Hugel, Seoul, Korea)
□	Nabota^®^/Jeuveau^®^ (Daewoong, Korea)

## Data Availability

Data available upon request to the authors.

## Supplementary Materials

The following supporting information can be downloaded at: https://www.mdpi.com/article/10.3390/toxins14050329/s1, Table S1: On-label and off-label indications for different BoNT-A products (full indication); Table S2: On-label indications for export-only Korea BoNT-A products (full indication).
