# Supplementary material for: An Update on the Cosmetic Use of Botulinum Toxin: The Pattern of Practice among Korean Dermatologists
Source: Toxins (Basel). 2022 May 4;14(5):329. doi: 10.3390/toxins14050329 (PMC9147646; doi:10.3390/toxins14050329)
Supplement: Supplementary file 1 [file toxins-14-00329-s001.zip › toxins-1698732-supplementary.pdf]

Supplementary Materials: An Update on the Cosmetic Use of Botulinum Toxin: The Pattern of Practice among Korean Dermatologists

Nark-Kyoung Rho, Kwang-Ho Han and Hei-Sung Kim

Table S1. On-label indications for different BoNT-A products available in Korea (full indication).

Commercial name	Manufacturer	Essential blepharospasm	Hemifacial spasm	Strabismus	Dynamic equinus foot deformity	Cervical dystonia	Post-stroke upper limb spasticity	Chronic migraine	Neurogenic bladder	Axillary hyperhidrosis	Horizontal forehead dynamic lines	Glabellar dynamic wrinkles	Lateral canthal dynamic wrinkles
Botox®/Vistabel®	Allergan (U.S.A.)	■		■	■	■	■	■	■	■		■	■
Botox®/Vistabel®50U	Allergan (U.S.A.)	■		■	■		■			■		■	■
Botulax®	Hugel (Korea)	■			■		■					■	■
Botulax®150U	Hugel (Korea)	■			■		■					■	■
Botulax®200U	Hugel (Korea)	■			■		■					■	■
Botulax®300U	Hugel (Korea)				■		■						
Botulax®50U	Hugel (Korea)	■			■		■					■	■
BTX-A®	Lanzhou (China)	■	■	■									
Coretox®	Medy-Tox (Korea)						■					■	
Dysport®/Azzalure®	Galderma (Switzerland)	■	■		■	■	■					■	
Innotox®	Medy-Tox (Korea)											■	
Liztox®100U	Huons (Korea)											■	■
Liztox®200U	Huons (Korea)											■	■
Liztox®50U	Huons (Korea)											■	■

Meditoxin®/Neuronox®	Medy-Tox (Korea)	■			■	■	■					■	■
Meditoxin®/Neuronox®150U	Medy-Tox (Korea)	■			■	■	■					■	■
Meditoxin®/Neuronox®200U	Medy-Tox (Korea)	■			■	■	■					■	■
Meditoxin®/Neuronox®50U	Medy-Tox (Korea)	■			■	■	■					■	■
Nabota®/Jeuveau®	Daewoong (Korea)	■					■					■	■
Nabota®/Jeuveau®150U	Daewoong (Korea)	■					■					■	■
Nabota®/Jeuveau®200U	Daewoong (Korea)	■					■					■	■
Nabota®/Jeuveau®25U	Daewoong (Korea)						■					■	
Nabota®/Jeuveau®50U	Daewoong (Korea)	■					■					■	■
Xeomin®/Bocouture®	Merz (Germany)										■	■	■
Xeomin®/Bocouture®50U	Merz (Germany)										■	■	■

■: On-label indication

Table S2. On-label indications for export-only Korea BoNT-A products (full indication).

Commercial name	Manufacturer	Essential blepharospasm	Hemifacial spasm	Strabismus	Dynamic equinus foot deformity	Cervical dystonia	Post-stroke upper limb spasticity	Chronic migraine	Neurogenic bladder	Axillary hyperhidrosis	Horizontal forehead dynamic lines	Glabellar dynamic wrinkles	Lateral canthal dynamic wrinkles
Bienox®	BNC Korea (Korea)											■	
Hitox®	BMI Korea (Korea)											■	
Inibo®	Inibio (Korea)											■	
Jetema The Toxin®100U	Jetema (Korea)											■	
Jetema The Toxin®200U	Jetema (Korea)											■	
Protoxin®100U	Protox (Korea)											■	
ReNTOX®100U	PharmaResearch (Korea)											■	
Tyemvers®	Chong Kun Dang (Korea)											■	

■: On-label indication
